# Notes and News

**Published:** 1895-06-08

**Authors:** 


### NOTES AND NEWS.

(COLLECTED AND COMMUNICATED.)

THE scheme to erect a medical institute for female students at St. Petersburg is being favourably entertained.

DR. BERTIN, a French doctor, asserts that experiment shows that the serum of the non-immunised horse has the same value in the treatment of diphtheria as that of the immunised animal.

MR. QUARRIER, the initiator of the movement to provide Scotland with hospitals for consumptives, has just received £1,000 for the purpose. He requires £60,000 for his object, £15,000 only of which has yet been received.

A NEW hospital has been opened at Redditch by Lady Windsor. It was erected from the proceeds of a bequest by Mr. Edwin Smallwood, and is called after him. The present accommodation provides for 20 patients.

AMONGST the many charities carrying on a most useful work in a quiet, steadily-progressing manner may be numbered the After Care Association for the relief of the insane discharged recovered from asylums. A patient regarded in the light of recovered by asylum authorities is most frequently in a condition far from suitable to resume his ordinary work in the world. To help such persons to do so is the object of the society. Patients are boarded out in cottage homes. The care of the inmates is entrusted to some lady in the neighbourhood of the home, the secretary of the association frequently visiting them. Local district secretaries, likely to prove most useful, are now being appointed in many districts. The association needs the generous support of the public financially, and is anxious to secure the warm co-operation and assistance of medical superintendents of asylums and Guardians of the poor so essential to a complete ministration on their part. The offices of the association are at Church House, Dean's Yard, Westminster.

MR. LEOPOLD DE ROTHSCHILD will preside at the festival dinner of the Metropolitan Hospital on Thursday, June 13th, to be held at the Hotel Metropole.

THE Vienna doctors are advocating a new health resort possessing springs whose waters resemble those of Carlsbad and Marienbad. The small town is called Kidze, and is situated in Bosnia.

A BILL has been brought forward in Illinois which provides for the free treatment of inebriates. To effect a cure the patient must consent to the arrangement, and the method of treatment is to be left at the discretion of the Court.

HER MAJESTY THE QUEEN has graciously become patron of the grand bazaar in aid of St. Mary's Hospital which is to be held at Portman Rooms on June 27th, 28th, and 29th, which the Princess of Wales will open, and at which the Duke and Duchess of York will be present.

THE experience up to date as to the efficacy of the anti-toxin treatment for diphtheria was discussed at the annual meeting of the American Medical Association. The verdict was in favour of the treatment, though the speakers exercised a wise caution and reserve in limiting their deductions strictly to definite facts.

A "HOSPITAL SATURDAY" collection at Sydney has just been accomplished for the second time, and turned out to be a complete success, the ladies, as before, working vigorously. Counting what has been collected for this movement at different warehouses, &c., during the year, the respectable total of £3,400 has been got together. Several minor sums have still to be received.

THE new wing opened at the Broomhill Home, Kirkintilloch, by the Duchess of Montrose last week will form another lasting memorial to the energy and interest shown in charitable undertakings by the ladies of Glasgow and the district. The new wing, costing £2,500, forms part of a sum of no less than £40,000 collected through the efforts of ladies, who organised bazaars and in other ways obtained the money.

THE Derbyshire Royal Infirmary is about to abolish the very objectionable proceedings which have attended the election of the president of the institution annually. The retiring president will no longer be required to nominate his successor, nor to subscribe £100 to the infirmary on his retirement. Both requisitions were unreasonable, and the management have done well to remove them.

THE local authorities of the Eastern District of Haddingtonshire have caused a caravan to be constructed to serve the district as an infectious hospital. It will be interesting to observe whether this original scheme will enhance or mitigate the usual "site" difficulty which attends the establishment of a permanent fever hospital. That the idea has much to recommend it for service in a scattered district there can be no doubt. The caravan is to cost £100, and be provided with a tent for the nurse or for cooking.

